# Turning Waste into Taste: Effective Upcycling of By-Products for Innovative Food Solutions^§^

**DOI:** 10.17113/ftb.63.02.25.8962

**Published:** 2025-06

**Authors:** Swapna Sree Meduri, Sujatha Mudawath, Prabhakar Butti, Soujanya Kanneboina, Sucharita Devi Tattepalli, Supraja Thoomati, Neela Rani Rathod, Aparna Kuna, Krishna Lavuri, Srinivasa Chary Darshanoju, Kanmani Kalaivanan

**Affiliations:** 1 Department of Food and Nutrition, Post Graduate and Research Centre, PJTAU, Rajendranagar, 500030 Hyderabad, India; 2 National Institute of Nutrition, 500007 Hyderabad, India; 3 Extension Education Component, All India Coordinated Research Project on Women in Agriculture, Professor Jayashankar Telangana Agricultural University, 500030 Hyderabad, India; 4 Food and Nutrition, MFPI-Quality Control Laboratory Rajendranagar, 500030 Hyderabad, India; 5 Institute of Rice Research (IRR), PJTAU, Rajendranagar, 500030 Hyderabad, India; 6 Department of Statistics and Mathematics, College of Agriculture, PJTAU, Rajendranagar, 500030 Hyderabad, India; 7 Department of Food Science and Nutrition, CSC & RI, TNAU, 625104 Madurai, India

**Keywords:** waste valorisation, aquafaba, coffee silver skin, propolis, by-products, sustainable development goals

## Abstract

Waste management in the food manufacturing sector has become one of the most challenging aspects globally owing to the generation of enormous quantities of by-products, such as peels, seeds and undesirable flesh at various stages of the processing chain. However, these plant by-products are rich in important compounds particularly polyphenols and bioactive substances that significantly affect human health and can be utilised in numerous sectors as new, low-cost and economical raw ingredients. The aim of this review paper is to discuss various methods of valorising food waste, concentrating on upcycling, aquafaba, coffee silver skin, propolis, wine lees and avocado waste. Food waste is a substantial global issue, with the potential to affect food security, environment and economy. Upcycling is highlighted as a means to tackle food waste by repurposing high-value by-products such as fruit and vegetable residues. Aquafaba, a vegan alternative to egg white, is produced from chickpeas and has various culinary applications. Coffee silver skin, a by-product of coffee production, contains bioactive compounds that can be extracted and used in functional foods. Propolis, a resinous substance collected by bees, is rich in bioactive compounds with health benefits. Wine lees, a by-product of winemaking, can be processed to extract phenolic compounds and produce value-added products. Avocado waste valorisation focuses on converting avocado by-products into valuable products for various industries. The sustainable valorisation of food waste offers numerous benefits, such as reducing waste output, generating revenue and promoting resource efficiency. Collaboration between stakeholders is essential to advance research and implement sustainable management practices for food waste valorisation to achieve the Sustainable Development Goals (SDGs). Challenges such as scaling-up, regulatory frameworks, logistics, food safety and environmental impact must be addressed to effectively valorise food waste.

## INTRODUCTION

The United Nations (UN) has set as one of its sustainable development goals to reduce food waste by half by 2030 (*1*). Food waste is the term used to refer to both food loss that occurs earlier in the food chain and waste of food that is intended for human consumption. A closer look at this key issue of sustainability reveals that in rich societies, most of the food waste is produced by consumers (*2*, *3*). Educating customers about sustainable food practices is therefore essential to reduce food waste in the future. Records show that consumers can reduce their emissions by 12 % if they avoid all food waste at home (*4*). Waste can be classified based on the industry that produces it into agricultural, farming, brewing, dairy, fattening, *etc.* (*5*). An environmentally friendly way to tackle food waste and shortages in the food sector is upcycling. Reducing debris and its negative effect on the environment is achieved by upgrading and recycling waste. Food security, both domestically and globally, is improved by repurposing nutrients from food waste (*6*).

The increasing need for renewable technologies in the food industry is supported by policies like United States Department of Agriculture (USDA) and United States Environmental Protection Agency (USEPA), aiming to reduce and recover food waste (*7*). In various food industries, one of the main problems is recycling of food waste streams. Worldwide, South Korea and Japan have been the leading countries in upcycling food industry waste (*6*). Without a doubt, one of the biggest industries that produces enough waste to negatively affect the environment is the food security sector (*6*). According to Mirabella *et al*. (*8*), 42 % of food waste in developed countries is caused by households, 39 % by food processing industries, 14 % by the food service industry (restaurants and catering) and the remaining 5 % by retail and distribution. These wastes are recognised as an important source of nutrients, particularly polyphenols and bioactive chemicals that have a major impact on human health, according to many studies conducted in recent years. However, since they are thrown out with the trash, it is necessary to retrieve them (*9*).

The Food and Agricultural Organization (FAO) of the United Nations estimated that food waste accounts for approx. 30 % of the world's food output (*10*). The Food Index Report states that over 930 million tonnes of sold food were wasted in 2019 (*11*). Melikoglu *et al.* (*12*) stated that “the amount of food that is wasted globally is enough to feed the whole world’s hungry population”. Furthermore, food waste is predicted to cost $310 billion in underdeveloped countries and $680 billion in industrialized countries, according to FAO (*13*). Creative solutions must be developed to transform the issue of food waste into an economic opportunity (*14*).

### Food wastage in India

Food wastage in India has reached alarming proportions. According to the FAO (*13*), millions of tonnes of food are wasted every year. In India, 40 % of the food produced ends up in the garbage, which is equivalent to 92 billion rupees annually (*12*). Despite high food production, food waste in India contributes to starvation among the population and India ranks 105th out of 127 countries in the 2024 Global Hunger Index with a score of 27.3 (*15*).

### The consequences of food waste

**Fig. 1:**
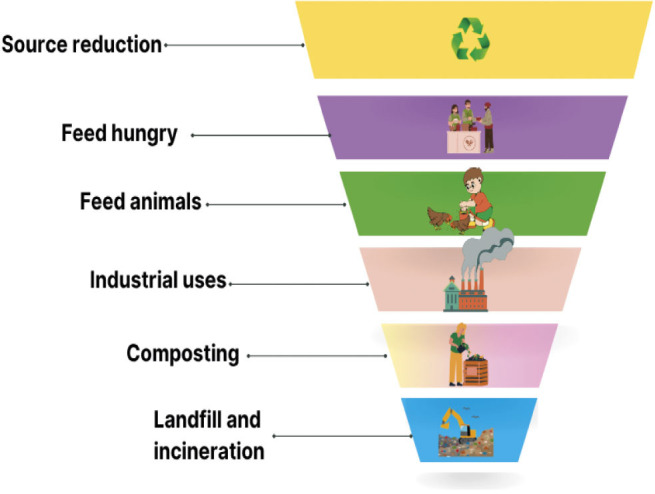
Food recovery hierarchy (*18*)

Food waste can be caused by a variety of variables, such as inefficient supply chains, production problems and customer behaviour (*16*). Farmers discard fruits and vegetables that are considered "abnormal," "ugly" or "inferior" because retailers have aesthetic standards for agricultural produce, according to Upcycled Food Association (UFA) (*17*). The US Environmental Protection Agency proposes a "food recovery hierarchy" to reduce food waste that starts at the source. Fig. 1 shows that alternative methods like composting and landfilling increase costs, so initiatives focusing on waste reduction at the beginning of the consumption cycle are recommended (*11*, *18*). According to Bhatt *et al.* (*16*), repurposing materials that would otherwise be thrown away and turning them into edible food could be a good way to address the issue of food waste.

### Difference between upcycling and recycling

Upcycling is the reuse of an object without causing degradation, while recycling involves transforming the original material into something different, requiring more energy, but both share environmental benefits (*19*).

Repurposed food is a relatively new food category (*20*) and is a solution to reduce food waste. The nutritional value of a product is increased through upcycling (*21*). According to Spratt *et al.* (*20*), the concept of upcycled food includes food items and ingredients that increase the use of food that would otherwise be wasted and have proven benefits for society and the environment. According to the UFA (*17*), the ingredients used for upcycled meals are sourced from places like farms, processing plants or retail outlets where they would not have been suitable for human consumption and are produced using traceable supply chains and are environmentally friendly. The production of food from recycled materials is an innovative technique that is recognised by many established and emerging food companies (*17*, *22*). The main goal of food recycling is to convert food that has been formally wasted into new sources of food (*14*). For this reason, food that has been upcycled is considered environmentally friendly. These foods reduce food waste during production (*16*). Because upcycled food has social advantages, consumers rated it higher than conventional food (*20*). Upcycling food has several benefits, including the ability to add value to the supply chain (*14*), increase profits and reduce dumping costs (*23*). It also increases efforts to conserve finite resources such as energy, labour, land, water and agrochemicals (*17*). The production of renewable food by food processing companies reflects the growing awareness of the environmental and economic benefits of these practices (*24*). Companies are concerned not only about the impact on the environment, but also about the impact on the future profitability of the company. This is the reason for changing and growing need to create new foods (*23*).

### Upcycled food trends in India

The demand for upcycled food is also growing In India. For example, Kocoatrait is a chocolate company in Chennai that uses recycled cocoa husk paper and other ingredients to make chocolate bars in a variety of flavours. Zero waste is the main goal of this organization. These repurposed foods are also served in commercial kitchens (*25*).

Indian restaurants such as SAGA, Ardana Modern Kitchen and Bar, Plural, and Taj Lands End are capitalising on the upcycling trend by serving traditional dishes created from various raw product parts, such as fish skin for chips and dehydrated vegetable peels for decoration. This strategy reveals how to reduce waste while maintaining taste and quality (*17*).

This review focuses on some upcycled products from different sectors of the food industry, their composition, the developed products, the use in different sectors and the nutritional benefits.

## AQUAFABA - VEGANS WHIP UP A SECRET WEAPON

More initiatives are being taken to mimic and replace animal supplies including meat, milk and eggs with plant-based food ingredients and products. This trend is linked to consumer preferences for wholesome, environmentally friendly food and the exponential growth of the vegetarian and vegan sector (*26*). Customers are willing to change their habits and address the issue of climate change by reducing their carbon footprint. This includes choosing plant-based foods over those derived from animals (*27*, *28*). Pulses contain 20–30 % protein high in lysine, such as lentils, chickpeas, faba beans, dried beans and dried peas. Consequently, pulses, edible dry seeds derived from legumes, can be quite helpful in replacing animal protein in the diet (*26*).

Recently, plant proteins have been increasingly favoured as potential substitutes for animal proteins (*29*). The food business and contemporary consumers both have a greater understanding of sustainability and health, which has sparked this interest. In the last 15 years, the production of legumes and pulses has increased significantly. India produced 13.75 million tonnes of chickpeas during 2021–2022 (fourth estimate) with a productivity of 12 600 kg/ha on 10.91 million ha of land (*30*). India's chickpea production accounts for almost half of the pulse production with a global market growing at a 7.0 % compound annual growth rate from USD 13.93 billion in 2022 to USD 14.9 billion in 2023. The market is forecast to reach USD 19.19 billion by 2027. India accounted for 73.46 % of chickpea production in 2020.

### Production and composition of aquafaba

Liquid aquafaba was obtained by steaming kabuli chickpeas in a pressure cooker for 30 min at a grain/water ratio of 1:3. After releasing the steam, the obtained liquid aquafaba is degassed for 20 min and dried with a spray or freeze dryer (*31*).

**Fig. 2:**
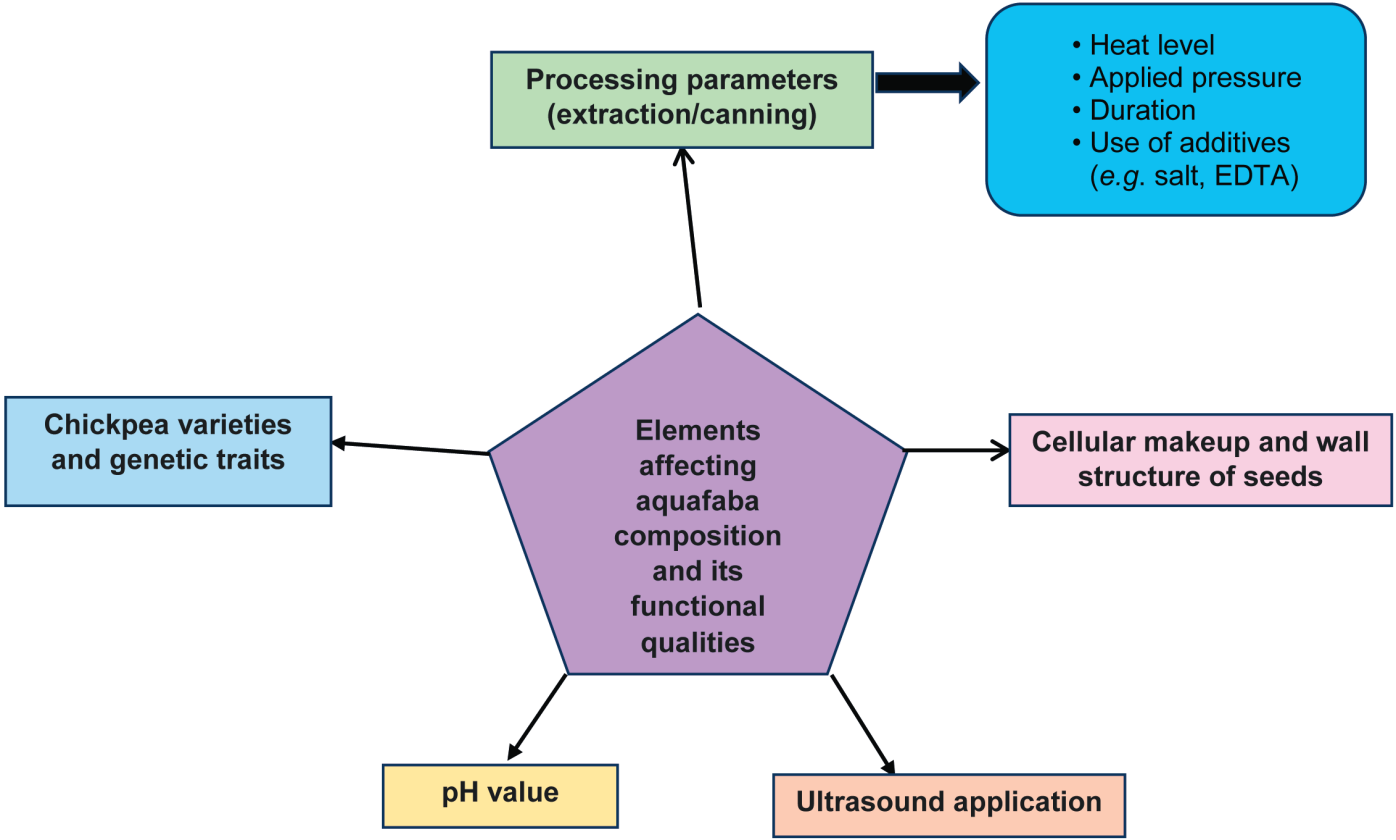
Factors affecting the quality and functional properties of aquafaba (*26*)

He *et al.* (*26*) optimised the production of aquafaba by varying the hydration and freezing times of dry chickpea seeds. The best quality was obtained after soaking for 16 h at 4 °C and cooking for 30 min followed by freeze and spray drying. The aquafaba content is influenced by three main factors: chickpea cultivar, seed and cell wall composition, extraction conditions and extraction temperature, pH, duration and pressure (Fig. 2) (*26*, *32*).

Aquafaba contains proteins and amino acids, but specific amino acid profiles for aquafaba are not well documented (*33*). Aquafaba has several advantages over other rheological additives (thickeners). It can replace multiple functions of eggs, such as foaming, emulsifying and gelling. It is also very stable, even after repeated freezing, thawing and heating. Aquafaba is environmentally friendly as it is a by-product of pulse processing, hummus production and frozen pulse production. The use of aquafaba instead of other additives such as plant proteins or hydrocolloids can reduce large amount of wastewater. Based on the physical factors mentioned above, the use of aquafaba in foods showed some problems, such as in sponge cake (110 mL aquafaba), where it mimicked the colour and texture of egg white cake but resulted in a sunken centre, large voids and reduced springiness (*34*). Mousse prepared with 80 g aquafaba, cream and sugar had comparable appearance and gloss to traditional mousse, without a beany odour, although it was perceived as slightly less sweet and smooth due to sodium and saponins (*35*). Gluten-free bread with 84 g aquafaba showed improved crumb softness and gas retention, but had less uniform pore distribution (*36*). Similarly, aquafaba in gluten-free crackers (147 g) improved colour, softness and moisture retention, but weakened the starch-protein network, resulting in higher breakdown (*37*). In mayonnaise (150 g aquafaba), the product had a deeper colour than the egg-based version, but was less accepted in terms of flavour and texture (*38*).

## COFFEE SILVERSKIN

Coffee is one of the most popular foods and the second most traded commodity after petrol (*39*). Coffee grounds and coffee silver husks are the most volatile residues in the world, containing high concentrations of tannin and caffeine. They can pose environmental hazards if disposed improperly. After the beans are separated, dried, ground and dehulled, coffee silverskin is obtained as a by-product (*40*, *41*).

The coffee processing industry should explore its by-products for environmental sustainability, as food waste can generate revenue, increase food security and combat hunger. Bioactive compounds like phenolic compounds, organic acids, proteins and polysaccharides can be extracted and reused as functional food ingredients. Novel extraction methods can preserve the quality of the active ingredients (*42*, *43*). Over 50 % of coffee fruit is discarded during processing, making it a potential source for incorporating coffee bioactive compounds into new functional foods (*39*).

Coffee roasting produces a by-product called coffee silverskin, which contains insoluble dietary fibre and high levels of antioxidant activity (*44*). Coffee silverskin consists of cellulose and hemicellulose, as well as significant amounts of glucose, monosaccharides, proteins and extractives, making it a valuable material for nutraceutical and cosmetic applications. On a dry mass basis, it contains 7.3 % moisture and is particularly rich in proteins, which make up 18.7 % of its composition. The fat content is between 2.2 and 3.8 %, while the ash content, which indicates its mineral content, is between 5 and 7 %. Carbohydrates form the largest content at 62.1 %, and it is also abundant in dietary fibre, particularly cellulose and hemicellulose, which together account for 16 to 23 % of the total content. This nutrient-rich profile emphasises the potential of coffee silver skin as a functional ingredient in both food and animal feed products (*39*).

The biological detoxification of coffee silverskin by solid-state fermentation leads to the production of phenolic compounds by fungal strains like *Aspergillus, Mucor, Penicillium* and *Neurospora* (*45*). The food industry is looking for ways to reduce sugar, fat and salt content in food while meeting consumer demands. Phytochemicals of coffee silverskin could be a functional ingredient to address issues like colour change and nutrient loss during food processing, potentially offering useful food applications (*46*). The researchers successfully developed high-quality bakery products like bread by adding coffee silverskin in combination with hydrogen peroxide to extend the shelf life (*47*). The scientists also describe a positive correlation between coffee silverskin and the water hydration, rheological properties, nutritional value and sensory properties of cookies (*48*). Minor phenolic compounds in coffee silverskin have antioxidant potential that may be a result of the Maillard reaction (*44*).

The results obtained by Ateş and Elmacı (*49*) showed that coffee silverskin treated with water can be used perfectly to replace 30 % of fat while maintaining physicochemical and sensory properties of the cake. However, the high fibre content of coffee silverskin makes it a low-calorie cake, which can extend the health benefits of the cake. The use of coffee silverskin as a substitute for wheat flour in the composition of cakes with better mechanical and colour properties has been reported (*49*). Coffee silverskin can be used in the development of functional beverages by promising fat reduction and mass control (*50*). Researchers reported that coffee silverskin is one of the functional ingredients for snacks, breakfast cereals, bread and biscuits and prepared a report proposing coffee by-products as a new food in the European Union (*51*, *52*). Coffee silver skin has been used in food formulations as a substitute and replacement in the bakery and beverage industry, which has been studied by various researchers (*47*, *49*, *50*).

## SPENT COFFEE GROUNDS

Instant coffee production involves treating coffee powder with steam or hot water, which produces a by-product called spent coffee grounds (SCG). Spent coffee grounds are produced in quantities up to 6 000 000 tonnes per year, usually as a result of instant coffee production (*40*). Despite their industrial importance, SCG have not been widely used. Solid-state fermentation (SSF) can minimise the amount of tannins and alkaloids in spent coffee grounds and thus reduce soil pollution. Despite the complexity and low economic feasibility of the extraction process, as well as the low triglyceride content of coffee oil, the lipids in spent coffee grounds have significant emulsifying properties (*39*).

Spent coffee grounds have a diverse and nutrient-rich composition that offers potential for various value-added applications. They contain 14.7 % minerals, which contribute to their inorganic nutrient content, and 17.4 % protein, which is a relatively high protein profile for a food by-product. The ash content is modest at 1.3 %, indicating a limited amount of residual inorganic matter. A notable feature of spent coffee grounds is their high polyphenol content of 17.8 %, which has an antioxidant effect and makes them attractive for use in functional foods, cosmetics or nutraceuticals. Additionally, polysaccharides make up the majority of the composition at 51.5 %, indicating a significant amount of complex carbohydrates that could be used in dietary fibre formulations or as a substrate in biotechnological processes (*53*).

Chlorogenic acid reduces body mass and waist circumference, promotes insulin secretion and controls blood pressure. It also reduces the accumulation of liver fat and increases lipase reactivity. Young people with degenerative diseases benefit from improved cognitive function by coffee. In mice with Alzheimer's disease, trigonelline improves brain function, memory and neuron activity. Melanoidins can trigger gene-protective pathways in several cell lines and have antioxidant and antibacterial properties. In addition, they support the fermentation of gut bacteria, the activation of antioxidant pathways and the population management of gut bacteria (*54*).

Spent coffee grounds have diverse applications because of their nutritional and functional properties. In the food industry, they improve dietary fibre content in bakery products, cookies and sponge cakes, and are used in fermented and distilled beverages. Coffee oil from spent coffee grounds is used as a butter substitute and flavouring in syrups. Spent coffee grounds also provide bioactive peptides and beneficial compounds like caffeine and chlorogenic acid. In cosmetics, they are valued for their anti-aging, sun protection and hydration effects. In addition to food and cosmetics, spent coffee grounds are also used in construction, bioenergy and as a material for biodegradable containers. However, variability, allergies, sanitation and high extraction costs continue to pose challenges (*55*).

## PROPOLIS

Propolis, a resinous substance produced by bees in their hives, has a potential use in food and health industries (*56*). The propolis market is predicted to expand at a compound annual growth rate (CAGR) of 4.90 % during the forecast period, from a value of USD 622.64 million in 2023.

Propolis, a plant-based food, is a complex substance with various biomolecules, including phenols, esters, terpenes, sugars and minerals. It contains varying amounts of components, including 45–55 % resins, 8–35 % wax, 5–10 % aromatic and essential oils, 5 % fatty acids, 5 % pollen and 5 % other organic components. The colour of propolis varies from greenish-yellow to brown, depending on maturity and origin. The characterisation of individual constituents like cinnamic acid and gallic acid can provide information about the health benefits of propolis (*57*).

**Fig. 3:**
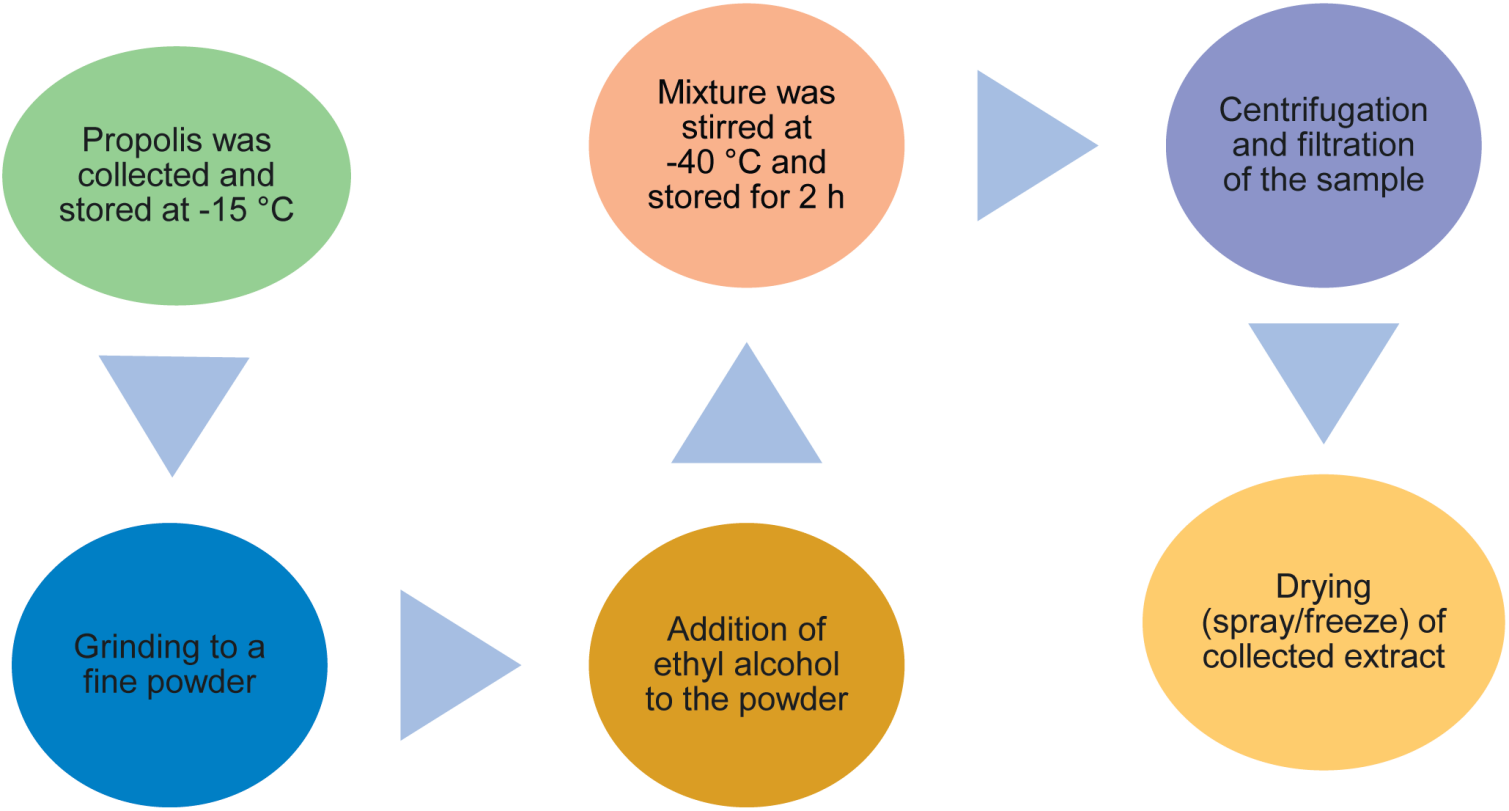
Flow chart of a procedure for preparation of propolis (*58*)

Propolis extract, which is obtained by extraction from crude propolis (Fig. 3), is widely used in food technology because of its significant antibacterial and antioxidant properties (*58*). When propolis extract is mixed with foods including fish, meat, milk, honey, fruit juice and beer, it reduces the total bacterial count, including *Staphylococcus* and *Listeria*, while protecting antioxidant food components. Soaking or washing fruits, vegetables and seafood in propolis extract reduces yeast, mould and bacterial infection, slows down ripening, minimises water loss, and preserves firmness, improving overall food quality. Furthermore, the addition of propolis extract to meat, fruit and vegetable packaging films significantly reduces microbial counts, including mesophilic and psychrotrophic bacteria and pathogens such as Enterobacteriaceae, thereby improving food preservation and safety in a wide range of food products (*59*, *60*). Coating and immersion techniques have been found to reduce the number of saprophytic and other microbiota in food (*61*).

Propolis, a healthy food, is categorised as a food supplement in different countries, including Brazil, the USA and the EU. However, because of differences in formulation and production site, the European Food Safety Authority (EFSA) has not yet released health statements for propolis on food labels, despite its potential health benefits (*62*).

## WINE LEES

The sediment produced during winemaking, *i.e.* fermentation, filtration, centrifugation and other additional processing during storage, is called wine lees, which constitutes 1.3–1.5 kg per litre of wine production (*63*). The components of wine lees, *i.e.* yeast, tartaric acid, phenolic compounds and other inorganic substances and enzymes, are of great importance in the food industry (*64*).

Wine lees, the sediment from wine fermentation, are nutritionally rich and promising for use in food, feed, cosmetics and agriculture. They contain 10.5–10.6 % ash, 21.2–21.9 % dietary fibre, 5.0–5.9 % lipids and 14.5–15.7 % proteins. Their polyphenol content (1.9–16.3 g/kg) offers antioxidant and antimicrobial benefits. The mineral content is significant, with high mass fractions of copper (1187 mg/kg), iron (84–1756 mg/kg) and potassium (17.6–158.1 g/kg) as well as zinc, phosphorus, magnesium, manganese and calcium. This composition shows that wine lees are a valuable resource for sustainable and functional applications (*63*). The phenolic compounds extracted from wine lees were studied (*65*). In this procedure, the preliminary treatment of wine less for conversion to powder for further use in food products and other studies was presented.

### Reuse of wine lees

**Fig. 4:**
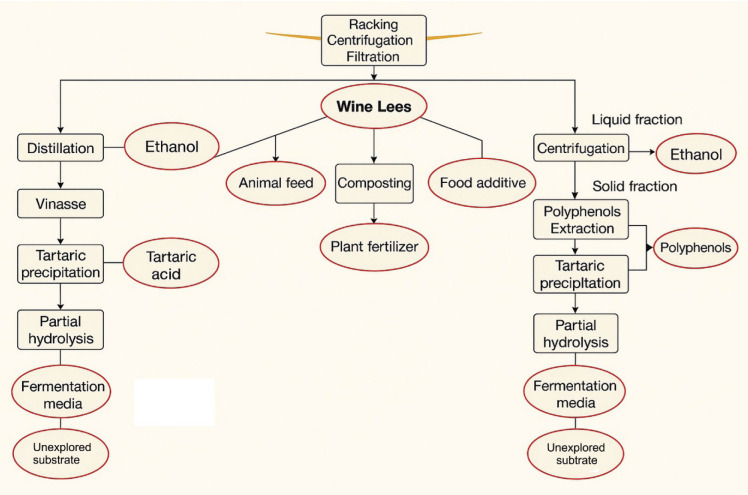
Various strategies for the valorisation of wine lees (*67*)

The wine lees show promising potential for several applications, such as replacing sulfur dioxide in winemaking with natural antioxidant and antimicrobial effects, with the stilbene-rich extracts particularly effective in preserving wine quality and aroma. As wine lees are abundant in fibre and protein, they have been successfully incorporated into value-added food products like cereal bars, ice cream and meat products to improve nutritional content, antioxidant activity and sensory appeal (*66*). The wine lees are used in the industry in different forms (Fig. 4), *e.g.* as animal feed, distillation additive, *etc.* (*67*).

### Health impacts

The polyphenols found in the by-products of winemaking can be divided into four main categories: (*i*) phenolic acids, which are found in hydroxybenzoic and hydroxycinnamic acids (*68*, *69*), (*ii*) flavonoids, which are divided into different classes, such as flavones, flavanons, flavonols, isoflavones, anthocyanins and proanthocyanidine (*70*), (*iii*) tannins, and (*iv*) stilbenes, which are primarily represented by *trans*-resveratrol and ε-viniferin (*66*). The positive effects of phenolic compounds, which have antioxidant, antimutagenic, anticarcinogenic and anti-inflammatory properties, have been the subject of several studies. In particular, studies have investigated the potential role of *trans*-resveratrol, epicatechin, quercetin, catechin and phenolic acids in the prevention of diabetes, cancer, heart disease, osteoporosis and neurological disorders (*71*). Their specific functions include the reduction of low-density lipoproteins (LDL), the resulting increase in high-density lipoproteins (HDL) and the prevention of platelet aggregation. In both healthy and ill individuals, phenolic compounds from grapes reduce the oxidation of plasma proteins by increasing the ability of serum to absorb oxygen radicals, promoting vasodilation (*72*) and lowering urinary F2-isoprostanes and other oxidative stress markers (*73*).

## AVOCADO

Avocado, a nutrient-rich fruit, is a popular choice because of its nutritional value (*74*). Mexico is the leader in the cultivation and export of avocado, with a production of 2.4 million tonnes in 2022. The fruit consists of pulp, seed and peel, which contain essential nutrients such as fibre, protein, healthy fats, vitamins C and E, phenolic compounds, carotenoids, chlorophyll and minerals (*75*). However, the increasing global demand for avocado production and processing leads to increased waste generation, causing environmental problems like greenhouse gas emissions, soil and water pollution and the attraction of pests. Urgent action is needed to address these environmental challenges (*76*, *77*).

Green valorisation can be used to turn avocado waste, which is rich in nutrients and compounds, into valuable products for the culinary, cosmetics or pharmaceutical industries (*78*). This strategy minimises the environmental impact and promotes resource efficiency by using the concepts of the circular economy. Composting, anaerobic digestion and the production of value-added products are sustainable methods of managing avocado waste that reduce adverse environmental effects and generate revenue (*79*-*81*).

Sustainable utilisation of avocado waste can improve farmers' lives and increase agricultural output. Promoting resource efficiency and reducing waste output through this method is in line with the sustainable development goals (SDGs). Focusing on the prospects and difficulties of setting up a biorefinery, the researchers explore the possibility of transforming avocado by-products into useful goods through biotechnological and environmentally friendly processes. The aim is to support sustainable practices in the avocado business and promote the avocado waste biorefinery as a sustainable way to treat processing by-products (*82*-*86*).

**Fig. 5:**
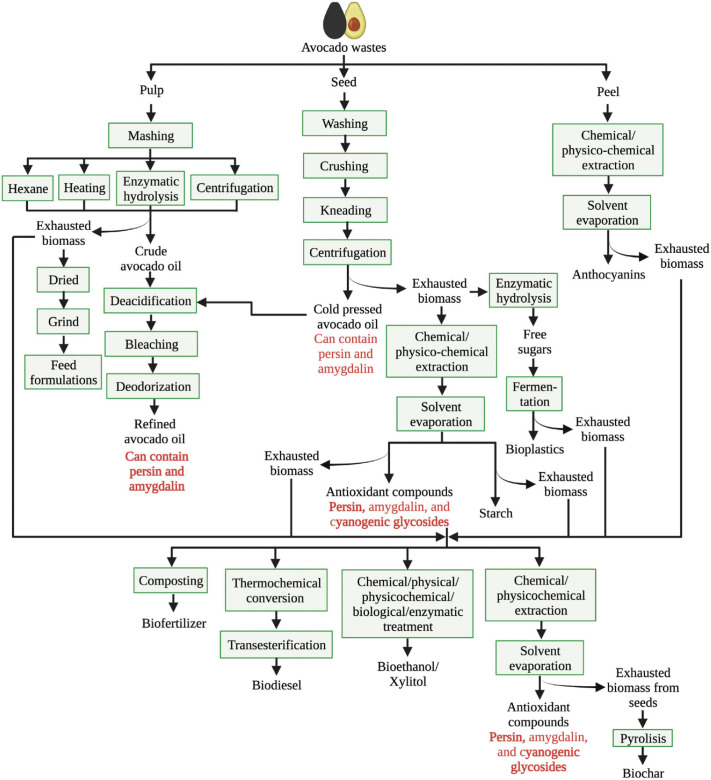
The primary procedure of a traditional avocado waste biorefinery (*75*)

Approaches to valorisation of avocado waste: in recent years, a number of methods have been developed to valorise avocado waste. One strategy uses methods like microwave-assisted extraction and supercritical fluid extraction to extract bioactive chemicals and antioxidants from the waste material, including polyphenols, flavonoids and carotenoids. The anti-inflammatory and antioxidant properties of these chemicals make them ideal candidates for use in the food and cosmetics industries. Another method is to use avocado waste, which has a high protein and fibre content, to produce animal feed for pigs, poultry and ruminants, as shown in Fig. 5 (*75*) and investigated in numerous studies (*81*, *82*, *87*-*90*). Persenone A is the most abundant acetogenin in avocado, accounting for 46 % in peel. These chemicals have been reported to have antioxidant effects by decreasing the formation of nitric oxide and superoxide but further studies are needed (*91*).

Avocado seeds can be used to develop ready-to-eat extruded snacks, with freeze-drying reducing potentially harmful compounds like persin and amygdalin to non-toxic levels. Cereal snacks enriched with 6–18 % avocado seed powder had negligible amounts of these compounds, while they had increased dietary fibre and polyphenol content, with 6 % leading to a fivefold increase in polyphenols. Snacks containing 20 % avocado seed flour and maize also had notable antioxidant activity. Avocado peel and seed extracts have proven effective, especially the ethanolic peel extract, which showed greater antimicrobial activity than ascorbic acid in mayonnaise. Additionally, avocado seeds are rich in starch with functional properties, a gelatinization temperature of 56–74 °C, a solubility of 19–20 % and a swelling power of 28–30 g/g, which emphasise their potential as an ingredient in functional food formulations (*75*).

The sustainable development goals (SDGs) can be significantly supported by the valorisation of avocado waste. By producing bio-products like food ingredients, biodegradable plastics, biogas and biofuels, waste production is reduced and resource efficiency is increased. Additionally, avocado waste can be used as a fuel for the production of bioenergy, supporting the use of renewable energy sources and reducing greenhouse gas emissions. Because jobs are created, this also strengthens employment and the local economy. Achieving the SDGs requires collaboration between government agencies, businesses and academic institutions (*92*-*94*).

Green valorization of avocado waste comes with a number of problems, including scale-up, regulatory framework, logistics and environmental impact (*95*). Handling Perishable waste must be handled carefully to prevent spoilage and to preserve its quality. Scaling up the process can be challenging since the composition, quality and quantity of waste varies greatly. Converting avocado waste into value-added products can consume energy and water resources, creating waste streams. It is essential to take sustainable measures to reduce these effects. There are still many unanswered questions regarding the authorisation procedures for food, cosmetics and pharmaceuticals within the legal framework for avocado waste valorisation (*75*).

## ADDRESSING CHALLENGES TO EFFECTIVELY VALORISE FOOD WASTE

The quantitative microbiological risk assessment (QMRA) approach has been refined with two new definitions: the appropriate level of protection (ALOP), which defines the level of health protection for foodborne pathogens, and the food safety objective (FSO), which defines the maximum hazard level in food at consumption to meet the ALOP. These definitions are complex and intended for use within the QMRA framework for global microbiological risk management. Policy makers and researchers recognise the need to engage society and promote consumer acceptance, particularly for novel foods produced from by-products. Addressing consumer concerns and preferences is crucial but requires further analyses and innovative approaches. The REPRO (Reducing Food Processing Waste project) consortium, funded by the European Commission, is transforming vegetable waste and cereal by-products into valuable food, feed and related ingredients (*96*). Using advanced bioprocesses and extraction technologies, the aim is to create safe, marketable products while ensuring compliance with regulations and consumer acceptance through risk assessments. This initiative targets the substantial waste from the EU vegetable and brewing industries and emphasises sustainability and innovation. Synthetic biology combined with advanced tools like tunable solvents (*e.g*. supercritical fluids) and microfluidic devices will drive the development of novel bioproducts such as biomaterials, bioenergy and biopharmaceuticals (Table 1; (*97*-*108*)). By integrating genetics into these technologies, biological engineers can create more functional biosystems for *in vitro* and *in vivo* applications, such as targeted drug delivery. With the growing interest in biofuels and environmental clean-up, supercritical extraction and synthesis techniques are expected to play a key role, including in biodiesel production and heavy metal removal. The future of these technologies depends on creativity at the intersection of science and engineering (*97*).

**Table 1:** Biotechnological recovery of compounds from waste materials in the food industry

Food industry	Type of waste material	Potential compound present	Biotechnological process for recovery	Result/yield	Potential application	Reference
Fruit and vegetable processing	Peels, seeds, pulp, stems, leaves	Pectin, cellulose, hemicellulose, sugars, phenolic compounds, vitamins, minerals	Enzymatic hydrolysis, solvent extraction, supercritical fluid extraction, fermentation	High yields of pectin, cellulose and phenolic compounds, ethanol, organic acids, enzymes, dietary fibre, biofuels, biogas	Food industry, pharmaceutical industry, biofuels, bioplastics	(*97*, *98*)
Dairy industry	Whey, milk permeate	Lactose, proteins, lipids, minerals	Membrane filtration, ultrafiltration, reverse osmosis, fermentation by lactic acid bacteria or yeast	High recovery of lactose and proteins (whey protein isolates, whey protein concentrate), lactic acid, ethanol, single-cell protein	Food and beverage industry, pharmaceutical industry, animal feed	(*99*, *100*)
Brewing industry	Spent grain, yeast	Proteins, carbohydrates, fibres, vitamins, minerals	Thermal drying, enzymatic hydrolysis, anaerobic digestion, fungal fermentation	High-protein animal feed, bioethanol, biogas, enzymes, fungal biomass	Biogas production, animal feed, food and beverage industry	(*101*, *102*)
Meat processing industry	Blood, bones, fat, offal	Proteins, lipids, minerals	Rendering, solvent extraction, enzymatic hydrolysis, anaerobic digestion	Oils, animal feed, fertilisers, biofuels, peptides, amino acids, gelatine, biofuels, biogas	Food industry, pharmaceutical industry, cosmetics industry, biofuels	(*103*, *104*)
Bakery industry	Bread crumbs, stale bread	Carbohydrates, proteins	Fermentation, anaerobic digestion	Ethanol, organic acids, biogas, animal feed	Biofuels, animal feed, food industry	(*105*, *106*)
Seafood industry	Fish waste, shellfish shells	Proteins, lipids, chitin, astaxanthin	Enzymatic hydrolysis, solvent extraction, chemical treatment	Fishmeal, omega-3 fatty acids, chitosan		(*107*, *108*)

Biotechnology plays a central role in the production of single-cell proteins (SCP), single-cell oils (SCO) and single-cell polysaccharides (SCP) as it can optimise microbial systems and increase production efficiency. Biotechnology is indispensable for the production of SCP, SCO and SCP and contributes to sustainable solutions for global challenges. It increases efficiency, enables resource utilisation and reduces environmental impact, making these bioproducts essential for future food, health and industrial needs (*109*).

## SINGLE CELL OILS

Microorganisms, including bacteria, fungi and yeasts, are being studied for their ability to produce single-cell oils (SCO) that could be used as a sustainable feedstock for biodiesel synthesis. These oleaginous microorganisms, which include yeasts such as *Lipomyces* and *Yarrowia*, can store 20–25 % of their biomass as lipids, which are often high in polyunsaturated fatty acids (PUFA). Lipid build-up is regulated by parameters such as the C/N ratio and nutrient depletion, and cultivation methods such as batch, fed-batch and continuous culture are used to increase production (*110*). SCO, which are used in animal feed, aqua feed and biodiesel, provide benefits such as regional independence and consistency in quality. However, high production costs, limited production capacity and competition from plant oils are barriers to commercialisation. Enzymatic techniques and waste substrates such as glycerol are being investigated to increase production efficiency, while research is focussing on the production of strains with high lipid accumulation and modified microbes (*111*). Biodiesel, a renewable and ecologically beneficial fuel, is typically produced from plant and animal oils, but energy demand and resource scarcity limit this strategy, making microbial oils a potential substitute. Extraction technologies such as solvent extraction make production easier, but customer acceptability and cost remain an issue. The "biorefinery concept", which uses low-cost substrates such as sugar cane and industrial fats, promises to reduce prices and increase SCO production for biodiesel, opening the door for a bio-based economy (*112*).

## SINGLE CELL PROTEINS

The current world population of 8.2 billion (2024) is expected to grow to 9.5 billion by 2050, leading to an increasing demand for food, especially meat. Currently, 370 million tonnes of beef are produced worldwide each year, but this figure will have to increase to 470 million tonnes by 2050. Traditional meat production is resource intensive, as almost 6 kg of plant protein is needed to produce 1 kg of meat protein. To solve this problem, single-cell protein (SCP), also known as microbial protein, has proven to be a promising solution. SCP is obtained from microorganisms such as algae, yeasts, fungi or bacteria and provides a protein-rich biomass. SCP was first developed in the 1960s as the "protein-from-oil process" and became widely recognised due to its promise to minimise dependence on traditional agricultural proteins (*113*). SCP has several advantages. It has fast production cycles, with microorganisms like bacteria and algae producing biomass within hours. It is environmentally friendly, requires less water and land, and has a smaller climatic impact than traditional agriculture. Furthermore, SCP can be genetically manipulated to improve amino acid content and utilise biodegradable industrial waste, reducing costs and environmental impact. However, there are also obstacles. The production of SCP is costly and requires strict sterilisation procedures. The high concentration of RNA can lead to gout and kidney stones as well as allergic reactions, poor digestion and taste or colouring problems. The production and quality of SCP depend on the microbial strains, environmental conditions (temperature, pH and light) and nutrient availability. To improve safety, the downstream manufacturing process includes isolation of the microbial cells by filtration or sedimentation, dissolution of the cell walls by mechanical, enzymatic or chemical processes and removal of nucleic acids (*114*). The final products, such as SCP concentrates, isolates and hydrolysates, are sterilised and dried. As the demand for sustainable protein sources increases, SCP present a viable option for meeting global nutritional demands, despite production and acceptability issues. Technological developments and further research can help solve these challenges, making SCP a feasible alternative in the future. Recombinant strains of nonconventional GRAS (Generally Regarded as Safe) yeasts and fungi should also be developed as part of the research. These strategies have a significant potential to improve the quality of meals, which is promising. It is necessary to thoroughly investigate the use of by-products from the food industry for the production of SCP (*115*).

## SINGLE CELL POLYSACCHARIDES

Single-cell polysaccharides (SCP) are microbial polymers produced by bacteria, fungi and algae and have a wide range of commercial and biological uses because of their diverse properties, which include thickening, gelling and stabilisation. Bacterial polysaccharides such as xanthan gum and curdlan, fungal polysaccharides such as pullulan and chitosan, and algal polysaccharides all have antioxidant and immunomodulatory properties (*116*). SCPs are highly sustainable since they can be produced from renewable substrates such as agricultural and industrial waste and are biodegradable, making them environmentally friendly alternatives to synthetic polymers. However, obstacles to large-scale commercialisation include high production costs, the need for optimised bioprocesses and strict regulatory requirements (*117*). Recent research has focused on the use of low-cost substrates and genetic engineering to increase yield and functionality, and establish SCPs as important contributors to a sustainable bioeconomy in the food, health and industrial sectors. With continued innovation, SCPs are expected to play an important role in long-term development (*118*).

Most of the by-products are reportedly derived from the food industry, which are rich in bioactive compounds and can be used in various industrial applications to promote health and nutritional benefits. A novel step in sustainable application is the use of these inexpensive by-products from the agri-food industry to create value-added products. Different patents are available in the field of food and biotechnology for the utilisation of by-products in the agro-food industry.

## CONCLUSIONS

The waste and by-products generated during the production of coffee, tea, fruit juices and alcoholic beverages have a vast potential for utilisation by industry. However, the large amounts of waste and by-products are underutilised. They can be made more valuable by conducting extensive research and development to identify and derive key bioactive molecules that will help in the creation of new value-added products with more affordable prices and health-promoting properties. Novel products that have the potential to reduce environmental pollution with excess by-products are needed.

Research is needed to standardise the conditions for aquafaba extraction and demonstrate its characteristics for consistent quality and functional properties. This is crucial for the innovation in chickpea waste recovery. Coffee silver skin has health-promoting properties for humans and contributes to sustainable health. Further studies are needed on spent coffee grounds as a food ingredient and the metabolic activity of their microbiota. Propolis needs to be standardised for therapeutic and medicinal purposes in dentistry, oral health and medicine. Further research is needed to recommend propolis as a dietary alternative for the treatment and prevention of chronic diseases. The influence of the components of wine lees on wine ageing needs further clarification. A study on the removal of tannins in avocado seed flour is recommended to improve flour quality.
